# Trichostatin A attenuates ventilation-augmented epithelial-mesenchymal transition in mice with bleomycin-induced acute lung injury by suppressing the Akt pathway

**DOI:** 10.1371/journal.pone.0172571

**Published:** 2017-02-24

**Authors:** Li-Fu Li, Chung-Shu Lee, Chang-Wei Lin, Ning-Hung Chen, Li-Pang Chuang, Chen-Yiu Hung, Yung-Yang Liu

**Affiliations:** 1 Department of Internal Medicine, Division of Pulmonary and Critical Care Medicine, Chang Gung Memorial Hospital and Chang Gung University, Taoyuan, Taiwan; 2 Department of Respiratory Therapy, Chang Gung Memorial Hospital, Taoyuan, Taiwan; 3 Chest Department, Taipei Veterans General Hospital, Taipei, Taiwan; 4 Institutes of Clinical Medicine, School of Medicine, National Yang-Ming University, Taipei, Taiwan; Seoul National University College of Pharmacy, REPUBLIC OF KOREA

## Abstract

**Background:**

Mechanical ventilation (MV) used in patients with acute respiratory distress syndrome (ARDS) can cause diffuse lung inflammation, an effect termed ventilator-induced lung injury, which may produce profound pulmonary fibrogenesis. Histone deacetylases (HDACs) and serine/threonine kinase/protein kinase B (Akt) are crucial in modulating the epithelial–mesenchymal transition (EMT) during the reparative phase of ARDS; however, the mechanisms regulating the interactions among MV, EMT, HDACs, and Akt remain unclear. We hypothesized that trichostatin A (TSA), a HDAC inhibitor, can reduce MV-augmented bleomycin-induced EMT by inhibiting the HDAC4 and Akt pathways.

**Methods:**

Five days after bleomycin treatment to mimic acute lung injury (ALI), wild-type or Akt-deficient C57BL/6 mice were exposed to low-tidal-volume (low-V_T_, 6 mL/kg) or high-V_T_ (30 mL/kg) MV with room air for 5 h after receiving 2 mg/kg TSA. Nonventilated mice were examined as controls.

**Results:**

Following bleomycin exposure in wild-type mice, high-V_T_ MV induced substantial increases in microvascular leaks; matrix metalloproteinase-9 (MMP-9) and plasminogen activator inhibitor-1 proteins; free radical production; Masson’s trichrome staining; fibronectin, MMP-9, and collagen 1a1 gene expression; EMT (identified by increased localized staining of α-smooth muscle actin and decreased staining of E-cadherin); total HDAC activity; and HDAC4 and Akt activation (*P* < 0.05). In Akt-deficient mice, the MV-augmented lung inflammation, profibrotic mediators, EMT profiles, Akt activation, and pathological fibrotic scores were reduced and pharmacologic inhibition of HDAC4 expression was triggered by TSA (*P* < 0.05).

**Conclusions:**

Our data indicate that TSA treatment attenuates high-V_T_ MV-augmented EMT after bleomycin-induced ALI, in part by inhibiting the HDAC4 and Akt pathways.

## Introduction

Acute respiratory distress syndrome (ARDS) is characterized by a severe inflammatory reaction and epithelial injury followed by fibroblast proliferation and extracellular matrix (ECM) deposition, which requires mechanical ventilation (MV) to provide life support [1, 2]. High-tidal-volume (high-V_T_) MV can initiate and propagate pulmonary neutrophil sequestration, enhanced alveolar–capillary membrane permeability, and the accumulation of protein-rich pulmonary edema; this ultimately leads to subsequent fibroproliferation and impaired gas exchange, a phenomenon termed ventilator-induced lung injury (VILI) [3, 4]. The epithelial–mesenchymal transition (EMT) process has been identified to mediate VILI-associated lung fibrosis by acquiring the phenotype of myofibroblasts differentiated from epithelial cells in both in vitro and in vivo studies [5–8]. Acute inflammation is followed by EMT, collagen deposition, and lung fibrosis through the production of profibrotic cytokines, including transforming growth factor (TGF)-β, plasminogen activator inhibitor (PAI)-1, and matrix metalloproteinase (MMP)-9 [8, 9]. Patients with ARDS who developed progressive lung fibrosis exhibit poor clinical prognosis [1, 2, 10]; therefore, strategies to ameliorate the fibroproliferative activity may enhance survival and improve quality of life.

Acetylation is one of the most crucial posttranslational modifications of histones that determines the structure and function of chromatin, and is hence related to gene transcription [11, 12]. Histone deacetylases (HDACs) are crucial posttranslational modifiers that remove acetyl groups from histones and transcription factors, and epigenetically regulate the expression of various genes [11, 12]. HDACs have been proven to be involved in fibrogenesis in various organs, including the lungs [13–17]. For example, Korfei et al. identified aberrant overexpression and activity of HDACs in the lungs of patients with idiopathic pulmonary fibrosis (IPF) [15]. Notably, HDAC4 is necessary for the TGF-β-induced fibroblast to myofibroblast transition because it inhibits histone acetylation [18, 19]. Furthermore, HDAC4 knockdown was reported to inhibit TGF-β signaling, indicating the essential role of HDAC4 in the epigenetic regulation of myofibroblast transdifferentiation in human lung fibroblasts [19].

Following acute inflammation, excessive reactive oxygen species (ROS) can induce damaged pulmonary epithelia to secrete proinflammatory and profibrotic cytokines that lead to imbalances between histone acetylation and deacetylation [20]. In rodents, PAI-1 and MMP-9 were shown to be essential cytokines for the recruitment of neutrophils (a primary source of ROS in acute lung injury [ALI]) and subsequent ECM remodeling in VILI pathogenesis [1, 9, 21]. However, the molecular mechanisms of HDACs, inflammatory-cytokines, and VILI-associated lung fibrosis remain unclear. HDAC inhibitors can act as effective anti-inflammatory or antifibrotic drugs by changing histone acetylation or suppressing the transcription factors [22–26]. Trichostatin A (TSA), a hydroxamic acid, has been identified as the histone deacetylase inhibitor with the most potential among all class I and class II HDACs [1]. Several in vitro studies have demonstrated that TSA can attenuate EMT by restoring epithelial cadherin expression in renal tubular epithelial cells, hepatocytes, lens epithelial cells, and airway epithelial cells [14, 17, 27, 28]. TSA has also been identified to suppress α-smooth muscle actin (α-SMA) expression and collagen production in rat hepatic stellate cells, rat skin fibroblasts, and primary human skin fibroblasts [29–31]. Furthermore, the prevention of ECM deposition and fibrotic scores has been achieved using TSA in the mouse models of bleomycin-induced skin fibrosis and pulmonary fibrosis, respectively [32, 33].

The activation of Akt is critical in mediating the differentiation of mesenchymal cells into smooth muscle cells [34]. A previous in vitro study demonstrated that Akt phosphorylation was modulated by HDAC4 in the regulation of TGF-β1-mediated α-SMA expression [19]. Additionally, Tan et al. showed that Akt plays a role in regulating bleomycin-induced EMT in mice [35]. In our previous study, we demonstrated that high-V_T_ ventilation-aggravated pulmonary fibrosis was dependent on the activation of the Akt pathway using an in vivo bleomycin mouse model [36]. However, the relationships between the Akt pathway and mechanical stretch-induced epigenetic programming remain unclear. In the present high-V_T_ MV-induced lung fibrosis model in mice pretreated with bleomycin, we first explored whether TSA could inhibit MV-augmented bleomycin-induced EMT and pulmonary fibrosis. Subsequently, we compared the effects of various tidal volumes of MV and examined the role of Akt kinase in the anti-inflammatory and antifibrotic effects of TSA on Akt-deficient mice. We hypothesized that following bleomycin-induced ALI, TSA administration can ameliorate high-V_T_ mechanical stretch-augmented EMT by suppressing the HDAC4 and Akt pathways.

## Materials and methods

### Ethics of experimental animals

Wild-type or Akt-deficient C57BL/6 mice, aged between 6 and 8 weeks, weighing between 20 and 25 g, were obtained from Jackson Laboratories (Bar Harbor, ME) and National Laboratory Animal Center (Taipei, Taiwan), as described in our previous study [36]. We performed the experiments in accordance with the National Institutes of Health Guidelines on the Use of Laboratory Animals. The Institutional Animal Care and Use Committee of Chang Gung Memorial Hospital approved the protocol (Permit number: 2015101201).

### Experimental groups

Animals were randomly distributed into 6 groups in each experiment: group 1, control, nonventilated wild-type mice without bleomycin; group 2, control, nonventilated wild-type mice with bleomycin; group 3, V_T_ 6 mL/kg wild-type mice with bleomycin; group 4, V_T_ 30 mL/kg wild-type mice with bleomycin; group 5, V_T_ 30 mL/kg Akt ^+/-^ mice with bleomycin; group 6, V_T_ 30 mL/kg wild-type mice with bleomycin and 2 mg/kg TSA administration. In each group, three mice underwent micro-computed tomography (micro-CT) and five mice underwent measurement for Evans blue dye (EBD) assay, lung edema, bronchoalveolar lavage (BAL) fluid total protein, malondialdehyde, PAI-1 and MMP-9 production, Masson’s trichrome stain, total collagen and hydroxyproline contents, collagen gene expression, immunofluorescence labeling, fibrosis scoring, Western blot, and immunohistochemistry assay.

### Ventilator protocol

We used our established mouse model of VILI, as previously described [8]. In brief, a 20-gauge angiocatheter was introduced into the tracheotomy orifice of mice and general anesthesia was maintained by regular intraperitoneal administration of zoletil 50 (5 mg/kg) and xylazine (5 mg/kg) at the beginning of experiment and every 30 min. Five hours of MV was applied for western blot, real-time polymerase chain reaction (PCR), PAI-1 and MMP-9 production, lung water, EBD, total collagen, collagen gene, free radicals, histologic staining analyses, and micro-CT imaging, based on our time course and previous studies [8, 36]. At the end of the study period, heparinized blood was taken from the arterial line for analysis of arterial blood gas, and the mice were sacrificed by exsanguination under zoletil and xylazine anesthesia to minimize suffering. The nonventilated control mice were anesthetized and sacrificed immediately.

### Bleomycin and trichostatin A administration

HDAC inhibitor (trichostatin A, Sigma, St. Louis, MO) 2 mg/kg was given intraperitoneally 1 h before ventilation based on our present and previous studies that showed 2 mg/kg inhibited HDAC activity [19]. The mice received a single dosage of 0.075 units of bleomycin in 100 μL of sterile normal saline solution intratracheally (Sigma, St. Louis, MO) and were ventilated 5 days after the administration of bleomycin [8].

### Immunofluorescence labeling

The lung tissues were paraffin embedded, sliced at 4 μm, deparaffinized, and stained according to the manufacturer’s instruction for an immunohistochemical kit (Santa Cruz Biotechnology, Santa Cruz, CA). Lung sections were incubated with primary rabbit anti-mouse antibodies of E-cad and α-SMA (1:100; New England BioLabs, Beverly, MA) and fluorescent secondary antibodies of FITC-conjugated affinity purified anti-goat (E-cad) and Cy3-conjugated anti-rabbit (E-cad and α-SMA) (1:1000; Santa Cruz Biotechnology, Santa Cruz, CA). Nuclear staining was performed using Hoechst solution (0.5 μg/mL; Sigma, St. Louis, MO). The fluorescence-labeled slides were subsequently examined using a Leica TCS 4D confocal laser scanning microscopy system (Leica, Wetzlar, Germany).

### Extraction of nuclear protein

The lungs (0.12–0.17 g) were homogenized in buffer A (lysis buffer) containing 50 mM KCl, 0.5% Igepal CA-630, 25 mM HEPES (pH 7.8), 1 mM PMSF, 2 μM leupeptin, 20 μg/mL aprotinin, 100 μM DTT and were subsequently incubated 5 min on ice, as previously described [37]. Cells were collected by centrifugation at 310 *g*, and the supernatant was decanted. The nuclei were washed in buffer A without Igepal CA-630, collected at 310 *g*, and resuspended in buffer B (extraction buffer) containing 500 mM KCl, 25 mM HEPES (pH 7.8), 10% glycerol, 1 mM PMSF, 2 μM leupeptin, 20 μg/mL aprotinin, and 100 μM DTT for 5 min on ice. The samples were subsequently frozen and thawed twice by dry ice and a 37°C water bath, rotating 20 min at 4°C, and centrifuged at 15,350 *g* for 20 min. The clear supernatant was collected, and nuclear protein concentration was measured by the Bradford method.

### Immunoblot analysis

The lungs were homogenized in 3 mL of lysis buffer (20 mM HEPES pH 7.4, 1% Triton X-100, 10% glycerol, 2 mM ethylene glycol-bis (β-aminoethyl ether)-N, N, Nˊ, Nˊ-tetraacetic acid, 50 μM β-glycerophosphate, 1 mM sodium orthovanadate, 1 mM dithiotreitol, 400 μM aprotinin, and 400 μM phenylmethylsulfonyl fluoride), transferred to eppendorf tubes and placed on ice for 15 min. Tubes were centrifuged at 15,350 *g* for 10 min at 4°C and supernatant was flash frozen. Crude cell lysates (Akt) and nuclear protein (HDAC 2 and HDAC4) were matched for protein concentration, resolved on a 10% bis- acrylamide gel, and electrotransferred to Immobilon-P membranes (Millipore Corp., Bedford, MA). For assay of Akt phosphorylation, and Akt, GAPDH, HDAC2, and HDAC4 total protein expression, Western blot analysis was performed with antibodies of phospho-Akt, Akt, GAPDH, HDAC2, and HDAC4 (New England BioLabs, Beverly, MA). Blots were developed by enhanced chemiluminescence (NEN Life Science Products, Boston, MA).

### Immunohistochemistry

The lungs were paraffin embedded, sliced at 4 μm, deparaffinized, antigen unmasked in 10 mM sodium citrate (pH 6.0), incubated with phospho-Akt primary antibody (1:100; New England BioLabs, Beverly, MA), and biotinylated goat anti-rabbit secondary antibody (1:100) according to the manufacturer’s instruction for an immunohistochemical kit (Santa Cruz Biotechnology, Santa Cruz, CA). The specimens were further conjugated with horseradish peroxidase-streptoavidin complex, detected with a diaminobenzidine (DAB) substrate mixture, and counterstained by hematoxylin. A dark-brown DAB signal, identified by arrows, indicated positive staining of phospho-Akt of epithelial cells, whereas shades of light blue signified nonreactive cells.

### Measurement of MMP-9 and PAI-1

At the end of the study period, the lungs were lavaged via tracheostomy with 20-gauge angiocatheter (sham instillation) 3 times with 0.6 mL of 0.9% normal saline. The effluents were pooled and centrifuged at 310 *g* for 10 min. Supernatants were frozen at -80°C for further analysis of the cytokines. MMP-9 and PAI-1 with lower detection limit of 0.014 ng/mL and 0.02 ng/mL were measured in BAL fluid by using commercially available immunoassay kits containing primary polyclonal anti-mouse antibody that was cross-reactive with rat and mouse MMP-9 and PAI-1 (MMP-9: Biosource International, Camarillo, CA; PAI-1: Molecular Innovations Inc., Southfield, MI). Each sample was run in duplicate according to the manufacturer’s instructions.

### Total HDAC activity assay

The nuclear protein extracted from lung tissue was incubated with assay buffer and *Color de Lys* substrate using a HDAC colorimetric assay/drug discovery kit (Enzo Life Sciences International, Inc., Plymouth Meeting, PA) and read on a standard microplate reader at 405 nm. Total HDAC activity was expressed relative to standard curve generated from 0–500 μM *Color de Lys* deacetylated standard.

### Real-time polymerase chain reaction

For isolating total RNA, the lung tissues were homogenized in TRIzol reagents (Invitrogen Corporation, Carlsbad, CA) according to the manufacturer’s instructions. Total RNA (1 μg) was reverse transcribed by using a GeneAmp PCR system 9600 (PerkinElmer, Life Sciences, Inc., Boston, MA), as previously described [8]. The following primers were used for real-time polymerase chain reaction (PCR): HDAC 4, forward primer 5′- CTGGCATCCCTGTGTCATTTG -3′ and reverse primer 5′- ACACAAGACCTGTGGTGAAC CTT -3′; and GAPDH as internal control, forward primer 5′- AATGCATCCTGCACCACC AA -3′ and reverse primer 5′- gtagccatattcattgtcata -3′ (Integrated DNA Technologies, Inc., Coralville, IA)[38]. All quantity PCR reactions using SYBR Master Mix were performed on an ABI Prism 7000 sequence detector PCR system (Applied Biosystems, Foster City, CA).

### Statistical evaluation

The Western blots were quantitated using a National Institutes of Health (NIH) image analyzer Image J 1.27z (National Institutes of Health, Bethesda, MD) and presented as arbitrary units. Values were expressed as the mean ± SD from at least 5 separate experiments. The data of EBD analysis, lung wet-to-dry weight ratio, BAL total protein, MDA, MMP-9, PAI-1, total collagen content, collagen gene expression, fibrosis score, and histopathologic assay were analyzed using Statview 5.0 (Abascus Concepts Inc. Cary, NC; SAS Institute, Inc.). All results of Western blots and real-time PCR were normalized to the nonventilated control wild-type mice with bleomycin treatment. ANOVA was used to assess the statistical significance of the differences, followed by multiple comparisons with a Scheffeˊs test, and a P value < 0.05 was considered statistically significant. BAL total protein, collagen assay, collagen gene 1a1expression, EBD analysis, histopathological grading of VILI, MDA, analysis of lung water, Masson’s trichrome stain, fibrosis scoring, and micro-CT were performed as previously described [8, 36].

## Results

### Reduction of VILI by TSA

**Fig 1:**
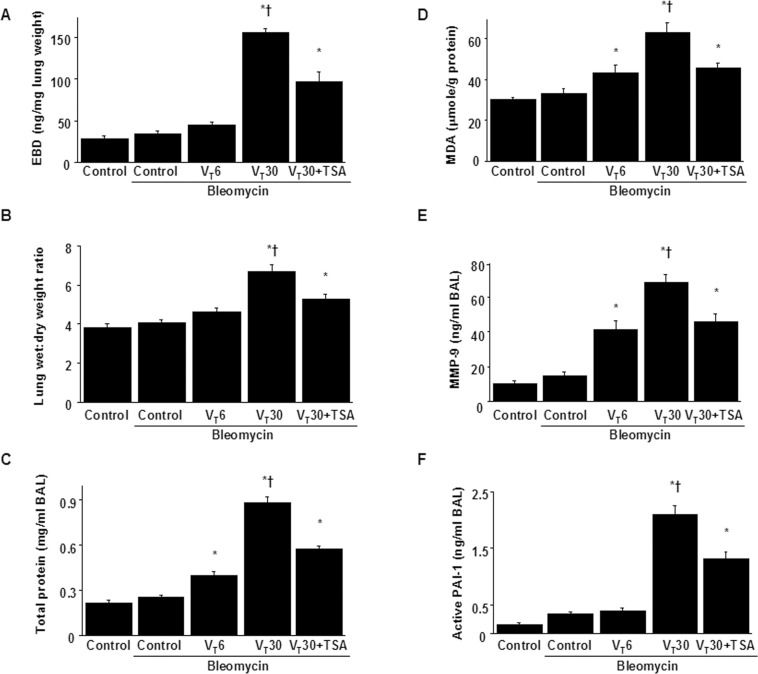
Trichostatin A inhibited lung stretch-induced microvascular leak, lung edema, oxygen radicals, MMP-9 and PAI-1 production. Five days after administering bleomycin, Evans blue dye analysis (A), lung wet-to-dry-weight ratio (B), BAL fluid total protein (C), MDA (D), MMP-9 (E) and PAI-1 (F) secretion in BAL fluid (E) were from the lungs of nonventilated control mice and mice ventilated at a tidal volume of 6 mL/kg (V_T_ 6) or 30 mL/kg (V_T_ 30) for 5 h with room air (n = 5 per group). TSA 2 mg/kg was given intraperitoneally 1 h before mechanical ventilation. ANOVA was used to assess the statistical significance of the differences, followed by multiple comparisons with a Scheffeˊs test, and a P value < 0.05 was considered statistically significant. * P< 0.05 versus the nonventilated control mice with bleomycin pretreatment; **†**P< 0.05 versus all other groups. BAL = bronchoalveolar lavage; EBD = Evans blue dye; MDA = malondialdehyde; MMP-9 = matrix metalloproteinase-9; PAI-1 = plasminogen activator inhibitor-1; TSA = trichostatin A.

High-V_T_ (30 mL/kg) and low-V_T_ (6 mL/kg) MV with room air was applied for 5 h to induce VILI in mice and elucidate the injurious effects of overstretch and treatment effects of intraperitoneally delivered TSA. The physiological conditions at the beginning and end of ventilation are listed in Table 1. The normovolemic statuses of the mice were maintained by monitoring their mean artery pressure. Additionally, the lung EBD microvascular leak, wet-to-dry weight ratio, and BAL fluid total protein were measured to determine the effects of high-V_T_ ventilation on microvascular leaks and lung edemas in VILI (Fig 1A–1C). Furthermore, the oxidant load and inflammatory cytokine levels were measured to determine the level of oxidative stress and amount of profibrogenic cytokines for fibroblasts in VILI (Fig 1D–1F). A higher wet-to-dry weight ratio and elevated levels of EBD, BAL fluid total protein, malondialdehyde (an aldehydic secondary product of lipid peroxidation), MMP-9, and PAI-1 protein were observed in the mice subjected to a V_T_ of 30 mL/kg compared with those subjected to a V_T_ of 6 mL/kg and the nonventilated control mice. Overall, TSA administration substantially suppressed the high-V_T_ MV-induced increase in lung inflammation.

**Table 1:** Physiologic conditions at the beginning and end of ventilation.

	Control without bleomycin wild-type	Control with bleomycin wild-type	V_T_ 6 mL/kg with bleomycin wild-type	V_T_ 30 mL/kg with bleomycin wild-type	V_T_ 30 mL/kg with bleomycin Akt^+/-^	V_T_ 30 mL/kg with bleomycin wild-type, TSA
PH	7.42±0.04	7.38±0.03	7.36±0.05	7.35±0.06	7.36±0.05	7.37±0.08
PaO_2_ (mmHg)	97.9±0.4	96.1±0.1	85.4±0.7*	76.5±1.4*†	86.9±1.2*	83.9±1.3*
A-aDO_2_ (mmHg)	3.3±0.2	4.6±0.1	13.1±0.2*	27.6±0.5*†	17.0±0.4*	19.9±0.5*
PaCO_2_ (mmHg)	39.1±0.3	39.4±0.2	41.2±0.3	36.7±0.8	36.8±1.4	36.9±1.8
MAP (mmHg)						
Start	85±0.8	83±0.6	83.4±1.2	82.4±1.5	83.2±1.5	82.7±1.6
End	83±0.7	81±0.4	79.6±1.5	73.7±4.4*†	78.8±1.9*	76.52±4.1*
PIP, mm Hg						
Start			9.8±1.1	23.7±1.4	23.4±1.9	23.5±1.0
End			11.9±0.8	28.5±2.1	26.8±1.7	27.4±2.5

At the end of the study period, arterial blood gases and mean arterial pressure were obtained from the nonventilated control mice and mice subjected to tidal volume at 6 mL/kg or at 30 mL/kg for 5 h (n = 10 per group). The normovolemic statuses of mice were maintained by monitoring mean artery pressure. Data are presented as mean ± SD. ANOVA was used to assess the statistical significance of the differences, followed by multiple comparisons with a Scheffeˊs test, and a P value < 0.05 was considered statistically significant.

* P< 0.05 versus the nonventilated control mice with bleomycin pretreatment.

**†** P< 0.05 versus all other groups.

A-aDO_2_ = alveolar-arterial oxygen gradient; Akt^+/-^ = serine/threonine kinase/protein kinase B -deficient mice; MAP = mean arterial pressure; PIP = peak inspiratory pressure; TSA = trichostatin A; V_T_ = tidal volume. The physiological data of the control groups were similar during the experiment and were used as the beginning data of ventilation.

### Suppression of MV-induced HDAC4 activation by TSA

**Fig 2:**
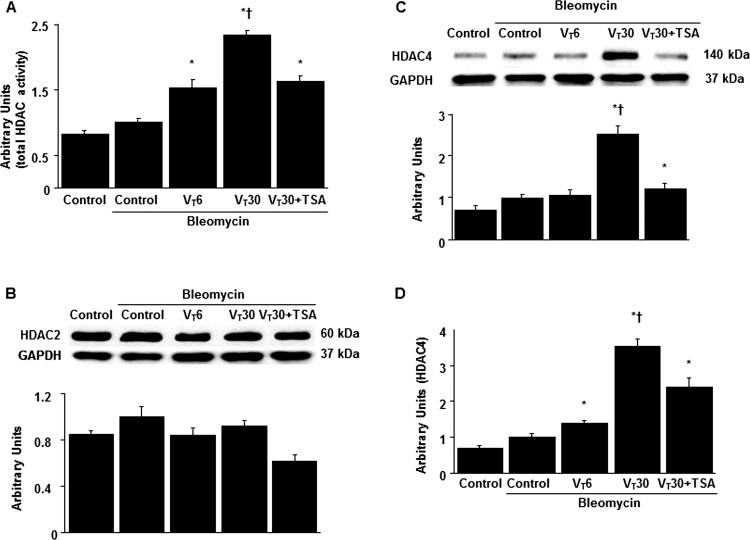
Trichostatin A reduced lung stretch-induced activation of HDAC4. (A) Five days after bleomycin treatment, total HDAC activity was from the lungs of nonventilated control mice and mice ventilated at a tidal volume of 6 mL/kg or 30 mL/kg for 5 h with room air (n = 5 per group). (B and C) Western blots performed using antibodies that recognize HDAC2, HDAC4, and GAPDH expression in lung tissue five days after bleomycin treatment were from nonventilated control mice and mice ventilated at a tidal volume of 6 mL/kg or 30 mL/kg for 5 h with room air (n = 5 per group). (D) Real-time polymerase chain reaction of HDAC4 mRNA and GAPDH mRNA expression in lung tissue after five days of bleomycin treatment were from nonventilated control mice and mice ventilated at a tidal volume of 6 mL/kg or 30 mL/kg for 5 h with room air (n = 5 per group). Arbitrary units were expressed as the ratio of HDAC2 to GAPDH, HDAC4 to GAPDH, and HDAC4 mRNA to GAPDH (n = 5 per group). TSA 2 mg/kg was given intraperitoneally 1 h before mechanical ventilation. ANOVA was used to assess the statistical significance of the differences, followed by multiple comparisons with a Scheffeˊs test, and a P value < 0.05 was considered statistically significant. * P< 0.05 versus the nonventilated control mice with bleomycin pretreatment; **†** P< 0.05 versus all other groups. HDAC = histone deacetylase; GAPDH = glyceraldehyde-phosphate dehydrogenase.

Because HDAC activation has been reported to play a crucial role in regulating stretch-induced EMT [18, 19, 28], we measured HDAC expression to investigate the role of HDAC signaling in our VILI model (Fig 2). The total HDAC activity, HDAC4 expression, and HDAC4 mRNA expression were increased in the mice subjected to a V_T_ of 30 mL/kg compared with those subjected to a V_T_ of 6 mL/kg and the nonventilated control mice. Notably, HDAC2 expression did not change significantly after MV. Moreover, the elevation of stretch-induced HDAC4 activity was considerably attenuated by TSA through pharmacologic inhibition.

### Reduction of collagen fiber production and collagen gene expression in mice ECM by TSA

**Fig 3:**
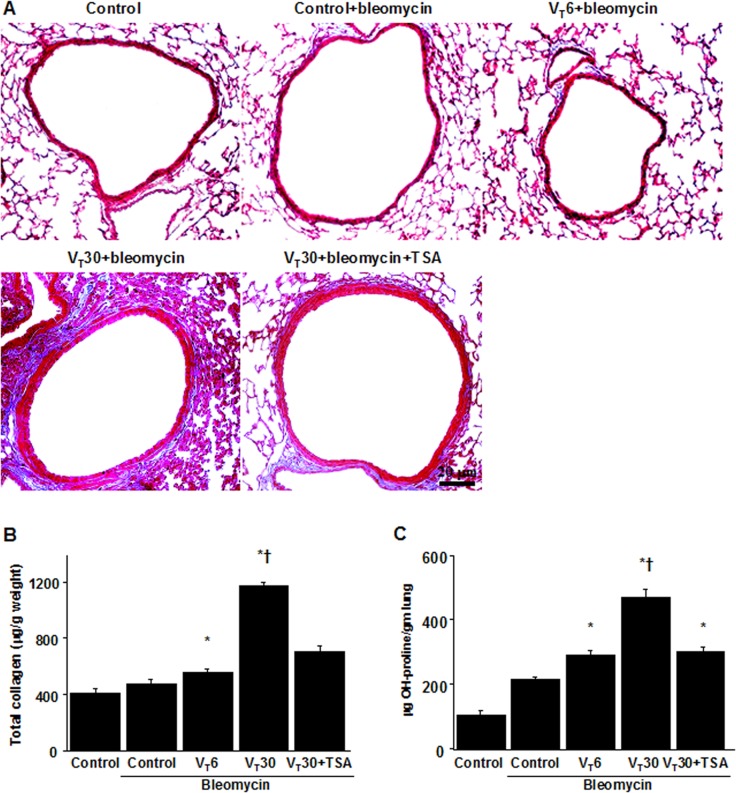
Trichostatin A reduced lung stretch-induced collagen production. Representative micrographs (x200) with Masson’s trichrome staining of paraffin lung sections (A), total collagen (B), and hydroxyproline (OH) (C) of lung tissue after five days of bleomycin administration were from nonventilated control mice and mice ventilated at a tidal volume of 6 mL/kg or 30 mL/kg for 5 h with room air (n = 5 per group). TSA 2 mg/kg was given intraperitoneally 1 h before mechanical ventilation. Scale bars represent 20 μm. ANOVA was used to assess the statistical significance of the differences, followed by multiple comparisons with a Scheffeˊs test, and a P value < 0.05 was considered statistically significant. * P< 0.05 versus the nonventilated control mice with bleomycin pretreatment; **†** P< 0.05 versus all other groups.

**Fig 4:**
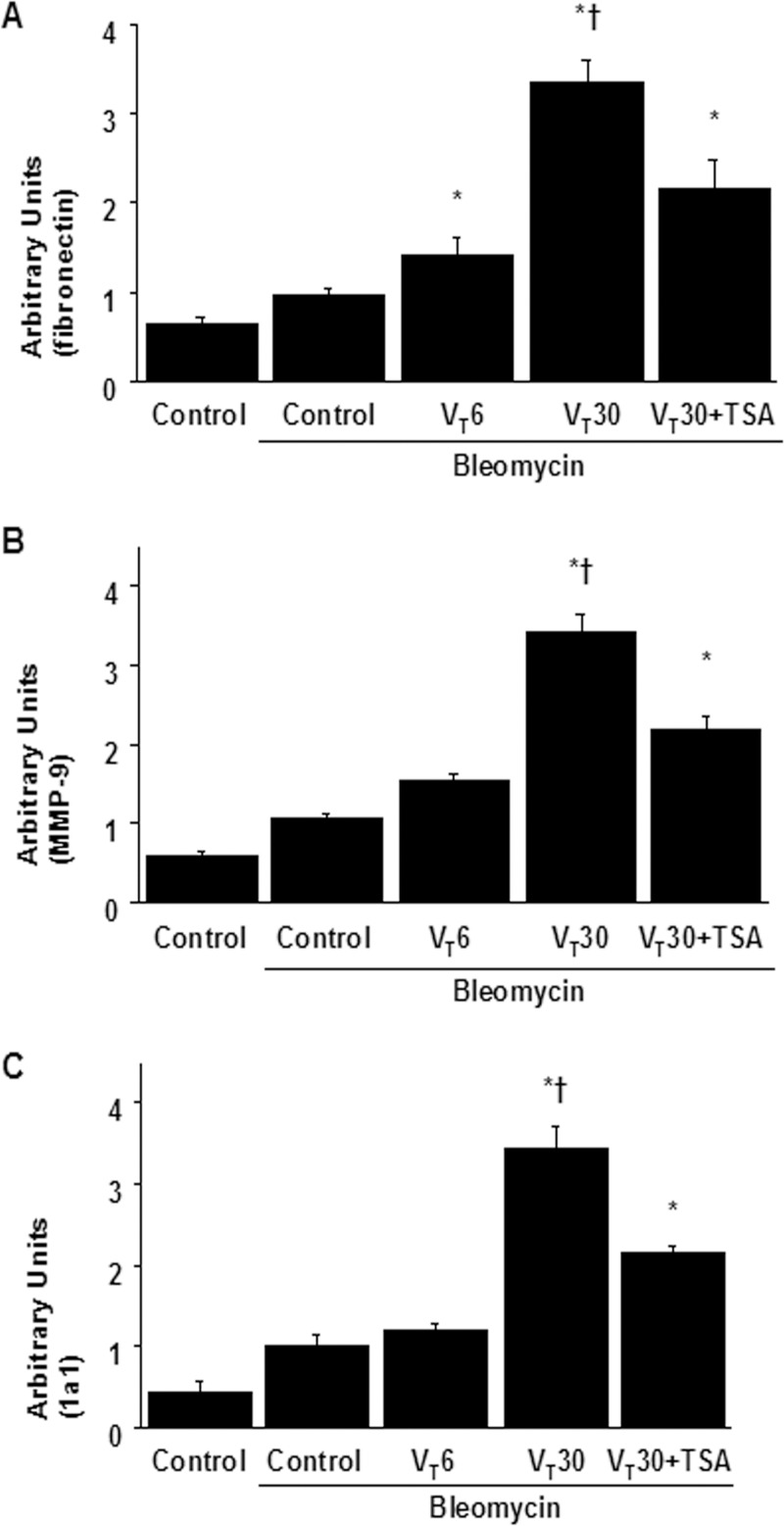
Trichostatin A suppressed lung stretch-induced collagen gene expression. After five days of bleomycin treatment, real-time polymerase chain reaction of fibronectin (A), MMP-9 (B), and 1a1 (C) mRNA expression were from the lungs of nonventilated control mice and mice ventilated at a tidal volume of 6 mL/kg or 30 mL/kg for 5 h with room air (n = 5 per group). Arbitrary units were expressed as the ratio of fibronectin, MMP-9, and 1a1 mRNA to GAPDH (n = 5 per group). TSA 2 mg/kg was given intraperitoneally 1 h before mechanical ventilation. ANOVA was used to assess the statistical significance of the differences, followed by multiple comparisons with a Scheffeˊs test, and a P value < 0.05 was considered statistically significant. * P< 0.05 versus the nonventilated control mice with bleomycin pretreatment; **†** P< 0.05 versus all other groups.

We also applied Masson’s trichrome staining to assess the effects of MV on the accumulated peribronchiolar and parenchymal collagen fibers. We observed increased collagen fibers in the ECM in the mice subjected to a V_T_ of 30 mL/kg compared with those subjected to a V_T_ of 6 mL/kg and the nonventilated control mice (Fig 3A). Measured quantitatively according to total lung collagen and hydroxyproline content, the increase in collagen fibers was substantially diminished through pharmacologic inhibition by TSA (Fig 3B and 3C). In addition, we used a real-time polymerase chain reaction to measure the expression of inflammation-associated collagens 1a1; fibronectin; and MMP-9. The expression of collagen 1a1, fibronectin, and MMP-9 was higher in the mice subjected to a V_T_ of 30 mL/kg compared with those subjected to a V_T_ of 6 mL/kg and the nonventilated control mice (Fig 4). Moreover, TSA administration appreciably reduced the expression of collagen 1a1, fibronectin, and MMP-9 associated with VILI.

### Inhibition of MV-induced fibrogenic markers by TSA

**Fig 5:**
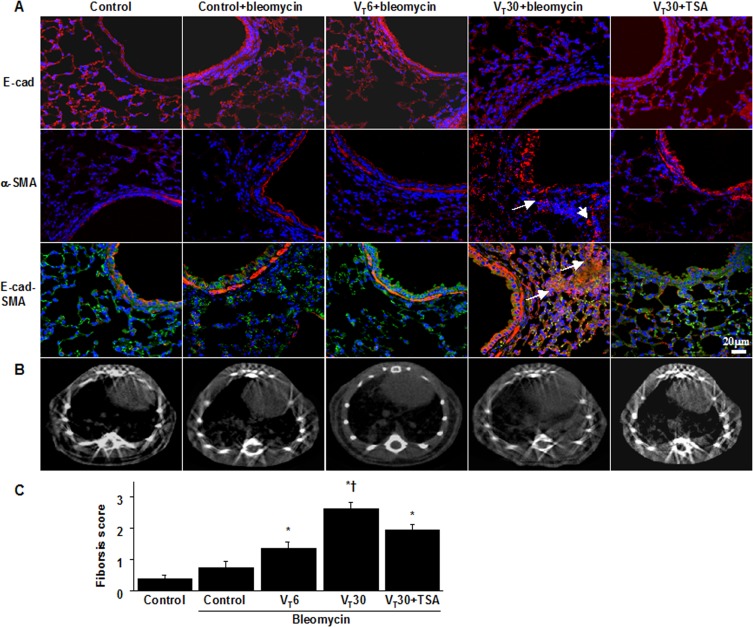
Trichostatin A attenuated lung stretch-induced fibrogenic biomarkers. Five days after bleomycin treatment, representative photomicrographs (x400) with E-cadherin (E-cad, red or bright green), α-smooth muscle actin (α-SMA, red), and Hoechst (blue) immunofluorescent staining of paraffin lung sections (A) (n = 5 per group), and micro-computed tomography imaging (B) (n = 3 per group) were from nonventilated control mice and mice ventilated at a tidal volume of 6 mL/kg or 30 mL/kg for 5 h with room air. TSA 2 mg/kg was given intraperitoneally 1 h before mechanical ventilation. Positive red and bright green staining in the lung epithelium and interstitium is identified by arrows (n = 5 per group). The fibrotic scoring (C) was quantified as the average number of 10 nonoverlapping fields in Masson’s trichrome staining of paraffin lung sections (n = 5 per group). Scale bars represent 20 μm. ANOVA was used to assess the statistical significance of the differences, followed by multiple comparisons with a Scheffeˊs test, and a P value < 0.05 was considered statistically significant. * P< 0.05 versus the nonventilated control mice with bleomycin pretreatment; **†** P< 0.05 versus all other groups.

We detected the expression of E-cadherin (an epithelial marker) and α-SMA (a mesenchymal marker) through immunofluorescent staining, which revealed the cell types involved in the MV-induced EMT (Fig 5A). Moreover, we examined the effects of TSA administration in attenuating EMT. Overall, the mice subjected to a V_T_ of 30 mL/kg experienced downregulation of E-cadherin and upregulation of α-SMA in their bronchiolar epithelia and peribronchiolar lung parenchymas, indicating a phenotype transition from epithelial cells to myofibroblasts. TSA also further increased the expression of E-cadherin and reduced the expression of α-SMA. In addition, we employed micro-CT, a primary imaging technique that monitors lung fibrogenesis in vivo, to examine the damaged lung structure [39]. Evidence of pulmonary fibrogenesis and damage, including increased reticular opacities, honeycombing, traction bronchiectasis, and ground glass opacities in the lower lungs, was aggravated in the mice ventilated at a V_T_ of 30 mL/kg compared with those subjected to a V_T_ of 6 mL/kg and the nonventilated control mice (Fig 5B). To further determine the effects of MV on ECM deposition, we performed fibrosis scoring through Masson’s trichrome staining (Figs 3A and 5C). Peribronchiolar ECM accumulation was elevated in the mice subjected to a V_T_ of 30 mL/kg compared with those subjected to a V_T_ of 6 mL/kg and the nonventilated control mice. Additionally, TSA administration substantially reduced the fibrosis score of ventilation-induced lung fibrogenesis.

### Suppression of the MV-induced Akt pathway by TSA and Akt heterozygous knockout

**Fig 6:**
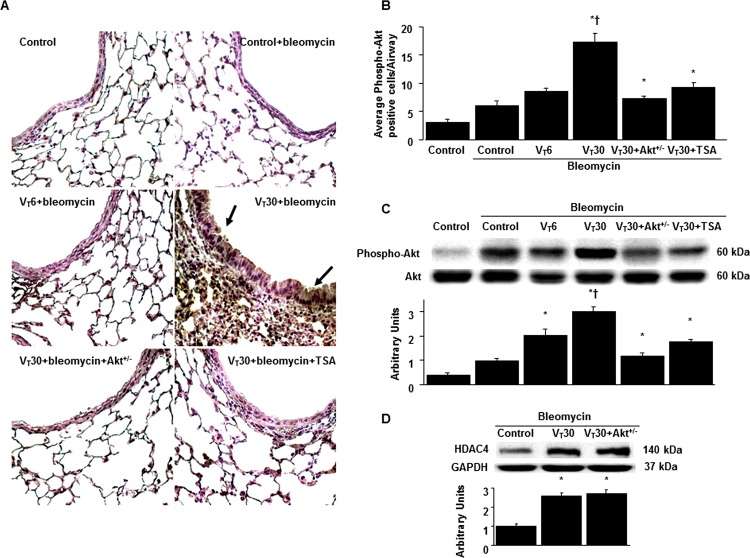
Trichostatin A and Akt heterozygous knockout abrogated lung stretch-induced Akt phosphorylation. (A and B) Representative micrographs (x400) with phosphorylated Akt staining of paraffin lung sections and quantification 5 days after administering bleomycin were from nonventilated control mice and mice ventilated at a tidal volume of 6 mL/kg or 30 mL/kg for 5 h with room air (n = 5 per group). (C and D) Western blots were performed using an antibody that recognize the phosphorylated Akt expression and antibodies that recognize total Akt, HDAC4, and GAPDH expression in lung tissue five days after bleomycin treatment from nonventilated control mice and mice ventilated at a tidal volume of 6 mL/kg or 30 mL/kg for 5 h with room air (n = 5 per group). Arbitrary units were expressed as the ratio of phospho-Akt to Akt and HDAC4 to GAPDH (n = 5 per group).TSA 2 mg/kg was given intraperitoneally 1 h before mechanical ventilation. ANOVA was used to assess the statistical significance of the differences, followed by multiple comparisons with a Scheffeˊs test, and a P value < 0.05 was considered statistically significant. * P< 0.05 versus the nonventilated control mice with bleomycin pretreatment; **†** P< 0.05 versus all other groups. Akt^+/-^ = serine/threonine kinase-protein kinase (Akt)-deficient mice.

Because Akt activation has been reported to regulate stretch-induced EMT [36, 40], we measured Akt phosphorylation to examine the role of the Akt pathway in our VILI model (Fig 6). The positive immunohistochemical staining for Akt in the airway epithelial cells of the mice subjected to a V_T_ of 30 mL/kg was attenuated in the Akt-deficient mice, and pharmacologic inhibition was observed with TSA treatment (Fig 6A and 6B). Consistent with the immunohistochemical results, Western blot analyses revealed increased Akt phosphorylation in the mice subjected to a V_T_ of 30 mL/kg compared with those subjected to a V_T_ of 6 mL/kg and the nonventilated control mice. However, total Akt nonphosphorylated protein expression did not change significantly. Moreover, the MV-induced phospho-Akt activation was markedly reduced by Akt heterozygous knockout and TSA administration (Fig 6C). However, the increased HDAC4 expression after MV was not attenuated in the Akt-deficient mice, suggesting that HDAC4 is the upstream regulator of Akt signaling involved in ventilation-induced lung fibrogenesis (Fig 6D).

### Inhibition of MV-induced lung inflammation and EMT by Akt heterozygous knockout

**Fig 7:**
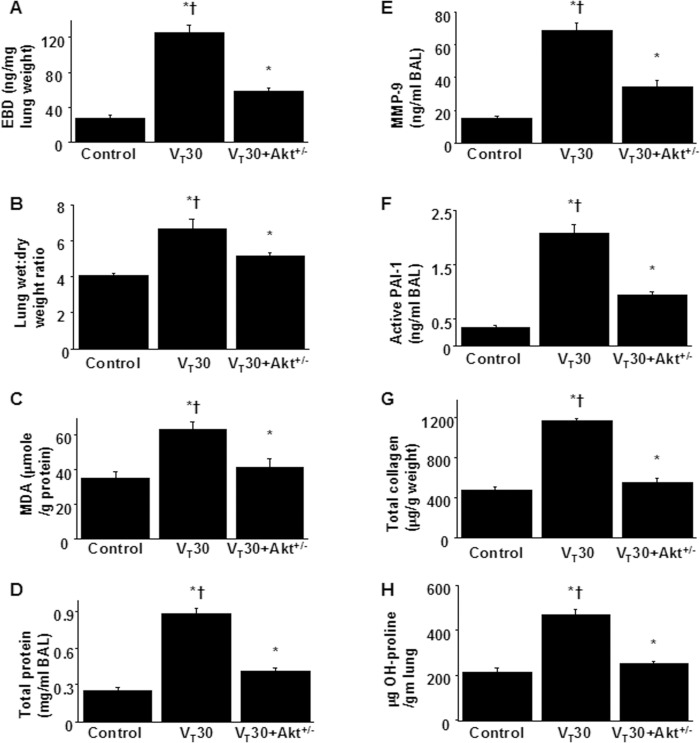
Inhibition of lung stretch-induced lung inflammation and collagen production by Akt heterozygous knockout. Five days after administering bleomycin, Evans blue dye analysis (A), lung wet-to-dry-weight ratio (B), BAL fluid total protein (C), MDA (D), MMP-9 (E) and PAI-1 (F) secretion in BAL fluid, total collagen (G), and hydroxyproline (OH) of lung tissue (H) were from the lungs of nonventilated control mice and mice ventilated at a tidal volume 30 mL/kg for 5 h with room air (n = 5 per group). ANOVA was used to assess the statistical significance of the differences, followed by multiple comparisons with a Scheffeˊs test, and a P value < 0.05 was considered statistically significant. * P< 0.05 versus the nonventilated control mice with bleomycin pretreatment; †P< 0.05 versus Akt^+/-^ group.

**Fig 8:**
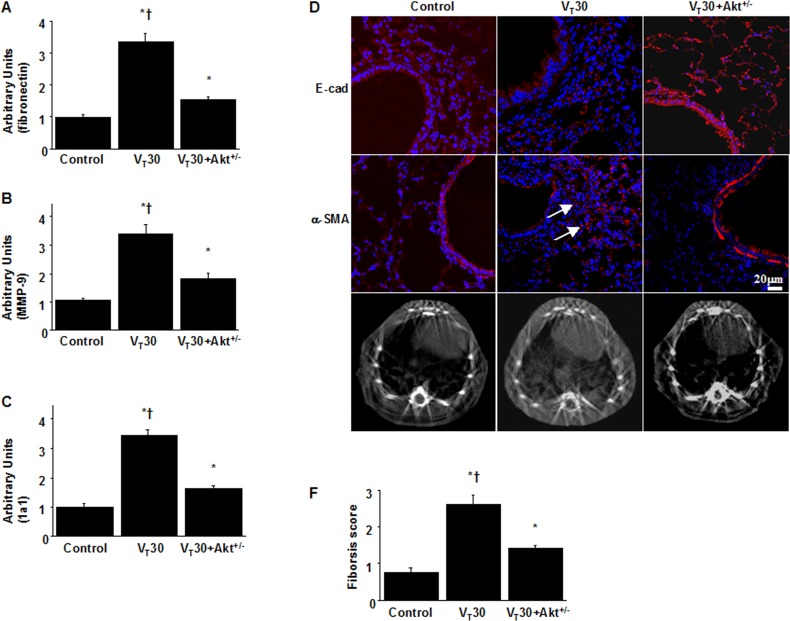
Reduction of lung stretch-induced collagen gene expression and epithelial-mesenchymal transition in Akt-deficient mice. After five days of bleomycin treatment, real-time polymerase chain reaction of fibronectin (A), MMP-9 (B), and 1a1 (C) mRNA expression were from the lungs of nonventilated control mice and mice ventilated at a tidal volume of 30 mL/kg for 5 h with room air (n = 5 per group). Arbitrary units were expressed as the ratio of fibronectin, MMP-9, and 1a1 mRNA to GAPDH (n = 5 per group). Representative photomicrographs (x400) with E-cad (red), α-SMA (red), and Hoechst (blue) immunofluorescent staining of paraffin lung sections (D) (n = 5 per group), and micro-computed tomography imaging (E) (n = 3 per group) five days after administering bleomycin were from nonventilated control mice and mice ventilated at a tidal volume of 30 mL/kg for 5 h with room air. Positive red staining in the fibroblasts is identified by arrows (n = 5 per group). The fibrotic scoring (F) was quantified as the average number of 10 nonoverlapping fields in Masson’s trichrome staining of paraffin lung sections (n = 5 per group). Scale bars represent 20 μm. ANOVA was used to assess the statistical significance of the differences, followed by multiple comparisons with a Scheffeˊs test, and a P value < 0.05 was considered statistically significant. * P< 0.05 versus the nonventilated control mice with bleomycin pretreatment; **†**P< 0.05 versus Akt^+/-^ group.

Finally, we examined whether the beneficial effects of TSA administration were mediated through the Akt pathway in Akt-deficient mice. Overall, the effects of high-V_T_ lung stretching on increases in lung EBD leak; BAL fluid total protein; oxidative stress; MMP-9 and PAI-1 protein production; mRNA expressions of collagen 1a1, fibronectin, and MMP-9; fibrogenic markers; and fibrosis scoring in the mice subjected to a V_T_ of 30 mL/kg were significantly attenuated in the Akt-deficient mice (*P* < 0.05; Figs 7 and 8). Furthermore, the gas exchange (alveolar–arterial oxygen gradient) in mice receiving a V_T_ of 30 mL/kg was considerably improved by TSA treatment and in Akt heterozygous knockout (Table 1).

Because no statistically significant differences were observed between the wild-type and Akt-deficient nonventilated control mice, both with and without bleomycin, the data are not presented in this paper.

**Fig 9:**
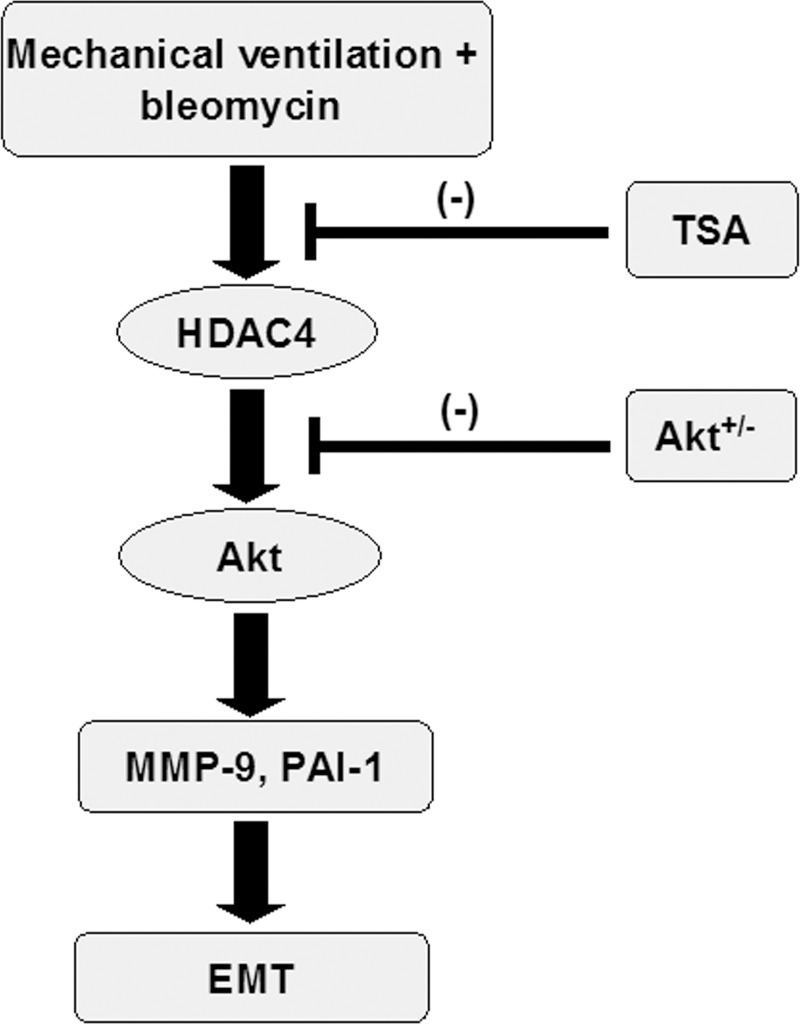
Schematic figure illustrating the signaling pathway activation with mechanical ventilation. Bleomycin-induced augmentation of mechanical stretch-mediated cytokine production and EMT were attenuated by the administration of trichostatin A and with Akt heterozygous knockout. Akt^+/-^ = Akt-deficient mice; EMT = epithelial-mesenchymal transition; HDAC = histone deacetylase; MMP-9 = matrix metalloproteinase-9; PAI-1 = plasminogen activator inhibitor-1; TSA = trichostatin A.

Collectively, our results demonstrate that TSA treatment can suppress MV-induced inflammatory responses and lung fibrogenesis, and improve gas exchange in the lung by inhibiting the HDAC4 and Akt pathways (Fig 9).

## Discussion

ARDS is a severe form of lung injury, characterized by increased capillary permeability, pulmonary edema, intense pulmonary inflammation, alveolar epithelial injury, and a high mortality rate [1, 2]. MV is essential to maintain adequate ventilation and gas exchange in patients with ARDS; however, MV by itself can trigger VILI or exacerbate the inflammatory response to fibrosis, resulting in VILI-associated lung fibrosis [3–6]. Accumulating clinical research has highlighted the importance of early identification of fibroproliferation in the lungs of patients with ARDS because a substantial percentage of ARDS survivors ultimately progressed to irreversible pulmonary fibrosis [1, 2]. Thus, early intervention targeting fibroproliferative activity, to ameliorate the progression to subsequent pulmonary fibrosis, may improve clinical outcomes.

In the present study, we demonstrated for the first time that the antifibrotic agent TSA can (1) mitigate the alveolar–capillary membrane leak and oxidative stress; (2) reduce inflammatory cytokines MMP-9 and PAI-1, collagen gene expression, and total collagen production; (3) attenuate the activation of HDAC enzyme specific for HDAC4, but not HDAC2, and suppress Akt phosphorylation; (4) alleviate the EMT process; and (5) ameliorate lung fibrosis as evidenced by improved pathological fibrotic scores, micro-CT images, and restored oxygenation indices in a murine model of MV-augmented bleomycin-induced pulmonary fibrosis. Furthermore, we observed HDAC4 regulated the downstream Akt signaling, and inhibition of HDAC4 and Akt mediates the beneficial effects of TSA in our animal model, which simulated the fibroproliferative response in clinical ARDS.

The fibroproliferative phase following acute exudative pulmonary inflammation has been previously considered a feature of late ARDS, characterized by fibroblast accumulation and ECM deposition in the injured lungs. However, clinical evidence has highlighted that increased fibroproliferative activity begins within 24 h of ARDS onset [2, 41]. Moreover, the development of pulmonary fibrosis has been associated with increased mortality, ventilator dependence, and restrictive lung disease, resulting in poor prognosis for both short- and long-term clinical outcomes [1, 2, 10]. The devastating role of MV in VILI-associated lung fibrosis has been confirmed in both in vitro and in vivo studies [3–8]. Specifically, high mechanical stresses disrupt the alveolar epithelial lining and lead to EMT activation in primary mammalian alveolar type II epithelial cells and cultured human lung epithelial cells [5, 6]. Additionally, the application of MV in animal models of ALI can induce epithelial injury and facilitate the development of pulmonary fibrosis by activating TGF-β1-induced EMT [5]. These study results suggest that MV can trigger EMT in diseased lungs and facilitate the progression of lung fibrosis. Hence, MV-induced EMT is considered to play a critical role in initiating and propagating VILI-associated lung fibrosis. In agreement with our previous findings, we demonstrated that high-V_T_ MV induced and augmented the destruction of the alveolar epithelial barrier and increased capillary permeability, oxidative stress, EMT, profibrotic cytokines regulating ECM remodeling, and excess fibronectin assembly in our VILI and MV-augmented bleomycin-induced pulmonary fibrosis animal models [7, 8, 36]. However, there are no specific pharmacological agents aimed at preventing early fibroproliferation in the lungs of patients with ARDS. The use of corticosteroids, a nonspecific anti-inflammatory agent, might shorten the MV duration; however, the steroids have the side effect of immunity suppression, and their clinical impact on improving ARDS survival remains unproven [42–44]. These uncertainties raise clinical urgency for investigating the molecular mechanisms involved in early fibroproliferation of ARDS and developing an effective therapy to inhibit EMT and lung fibrosis.

Human HDACs are divided into four classes according to their homology to yeast. Class I HDACs (HDAC 1, 2, 3, and 8) are primarily nuclear enzymes. Class II HDACs (HDAC 4, 5, 6, 7, 9, and 10) are expressed in a tissue-specific manner and shuttle between the nucleus and cytoplasm. Moreover, both class I and class II HDACs are zinc metalloenzymes that catalyze the hydrolysis of acetylated lysine residue. Class II HDACs have also been demonstrably crucial in the control of myofibroblast and smooth muscle functioning. Class III HDACs contain NAD^+^-dependent deacetylases (sirtuin 1–7), and class IV HDAC comprises HDAC11 only [13]. During acute inflammation, histone acetylation and deacetylation changes are involved in the induction of inflammatory cytokines, and subsequently, the mediation of EMT, fibroblast activation, and proliferation if inflammation persists and progresses to succeeding fibrogenesis [12, 20]. Tissue-specific HDACs are highly expressed and activated in inflamed tissues, epigenetically regulating the local production of PAI-1 and MMP-9 [45, 46]. Furthermore, PAI-1 and MMP-9 are relevant to neutrophil infiltration, EMT, fibroblast proliferation, and ECM remodeling in ALI [9, 21]. Overexpression and upregulation of HDACs, and increased levels of MMP-9 and PAI-1 in the lungs, have been identified in IPF clinical studies [9, 15, 46]. Among the various HDACs, HDAC2 and HDAC4 have been reported to be crucial in modulating cytokine signaling for immune responses [11, 25].

In the present study, we found that high-V_T_ MV augmented total HDAC activity, as well as PAI-1 and MMP-9 generation following bleomycin exposure. In particular, the pulmonary expression of nuclear HDAC4 significantly increased following MV-augmented lung fibrosis compared with HDAC2; this indicates that class II HDAC4 intensely translocated from the cytoplasm into the nucleus in the injured lungs, whereas class I HDAC2 was not altered. Thus, abrogation of HDAC4 activity by the HDAC inhibitor resulted in the exacerbated suppression of PAI-1 and MMP-9. Our results are in agreement with the findings of a previous in vitro study demonstrating that HDAC4 mediated the fibroblast to myofibroblast transition induced by TGF-β, and that HDAC4 knockdown inhibited TGF-β signaling in human lung fibroblasts [19]. On the basis of these results, the profibrotic effects of HDAC enzymes provide a promising therapeutic option in the management of VILI-associated pulmonary fibrosis using HDAC inhibitors.

TSA selectively inhibits the class I and class II mammalian HDACs and has been reported to have anti-inflammatory and antifibrotic activities both in vitro and in vivo [19, 33]. TSA can also inhibit the proliferation, migration, and differentiation to myofibroblasts and TGF-β-induced collagen secretion in primary human lung fibroblasts [19]. In animal models of bleomycin-induced pulmonary fibrosis, TSA treatment can prevent or inhibit pulmonary fibrosis identified by improving pathological fibrotic scores [33, 47]. In addition to its antineoplastic effects, TSA has been reported to produce antifibrotic effects through the inhibition of EMT in in vitro studies of human airway epithelia [23, 28]. Taken together, these findings triggered our interest to investigate the protective effects and molecular mechanisms of TSA on VILI-associated lung fibrosis in our animal model. Guo et al. demonstrated that TSA can blunt α-SMA and α1 type I collagen expression and ameliorate TGF-β-mediated myofibroblast differentiation by inhibiting HDAC4 in normal human lung fibroblasts [19]. Although these results are limited to in vitro conditions, they implicate the profibrotic role of HDAC4 in mediating the antifibrotic effects exerted by TSA.

In the present study, we demonstrated that TSA prevented EMT and increases in MMP-9 and PAI-1, collagen 1a1, total collagen, and lung fibrosis parameters through the inhibition of HDAC4 activation in vivo. Notably, TSA can also function as an autophagy-blocking agent in addition to its inhibitory effect on HDACs. Zhang et al. reported that TSA pretreatment improves MV-induced pulmonary permeability, lung edema, and neutrophilic inflammation, while also inhibiting stretch-induced autophagy activation in injured lungs [48]. These results are in agreement with our findings, and suggest that TSA provides beneficial effects on MV-associated lung damage and fibrosis because of the molecular mechanisms associated with modulating HDACs or autophagy-related genes.

The Akt pathway is required for TGF-β1-induced differentiation of mouse embryonic mesenchymal cells into smooth muscle cells characterized by α-SMA expression [34]. Specifically, Akt phosphorylation is elevated in fibroblasts isolated from the lungs of bleomycin-injured mice, and Akt inhibition attenuates the lung fibrinogenic activity [40]. One previous in vitro study demonstrated that Akt activation was enhanced by HDAC4 through the inhibition of protein phosphatase-mediated Akt dephosphorylation in the regulation of TGF-β1-mediated α-SMA expression in human lung fibroblasts [19]. Notably, Tan et al. reported that Akt was involved in the pathway regulating EMT in a mouse model of bleomycin-induced lung injury [35]. Additionally, in our previous study, we demonstrated that high-V_T_ MV augmented pulmonary fibrosis through the activation of Akt signaling using an in vivo bleomycin mouse model [36].

In the present study, we hypothesized that HDAC enzymes regulate the Akt pathway and lead to EMT and fibrosis. First, we proved that genetic downregulation of Akt can effectively reduce MV-augmented bleomycin-induced EMT and pulmonary fibrosis, as indicated by the diminished markers of vascular leakage and oxidative stress, decreased MMP-9 and PAI-1 production and Akt phosphorylation, decrement of total lung collagen, improved EMT profiles and pathological fibrotic alterations, and micro-CT images. Furthermore, Akt was demonstrated to be downstream to HDAC signaling (according to the finding that HDAC inhibition by TSA inhibited Akt activation), but HDAC4 expression was not suppressed in the Akt-deficient mice. Notably, we demonstrated that TSA can attenuate the EMT process, as evidenced by the increased localized epithelial marker (E-cadherin) and decreased localized mesenchymal marker (α-SMA) in our mouse model of MV-augmented bleomycin-induced pulmonary fibrosis, and that the beneficial effect is mediated through HDAC4-Akt signaling.

According to our review of the relevant literature, only one in vitro study by Guo et al. demonstrated that TSA can reduce TGF-β1-induced α-SMA expression associated with decreased Akt phosphorylation in normal human lung fibroblasts [19]. No in vivo study has investigated the effects and mechanisms of TSA on VILI-associated EMT and pulmonary fibrosis previously. Therefore, the present study is the first to demonstrate in vivo that TSA can mitigate EMT and subsequent pulmonary fibrosis in mice subjected to bleomycin exposure following MV, which simulates fibroproliferation in ARDS. In addition, we demonstrated that extensive epithelial injury results in increased alveolar capillary permeability and inflammatory parameters in our two-hit animal model. As revealed previously, epithelial cell injury can promote pulmonary fibrogenesis by acquiring a mesenchymal phenotype mediated by HDAC modification [28]. Mechanistically, we also demonstrated that intraperitoneal TSA administration can suppress the activation of HDAC4 and downstream Akt, and attenuate the subsequent pulmonary fibrosis by inhibiting EMT. Our results suggest that inhibition of EMT by TSA is critical to attenuate subsequent fibrogenesis in the setting of acute alveolar–epithelial injury.

Some limitations of our study must be discussed. The use of pan-HDAC inhibitor TSA may have suppressed other HDACs that were not investigated in this study. Previous studies have suggested that HDAC4 integrates with other HDACs, such as HDAC3 or HDAC5, to achieve the modification of chromatin structure and function [49, 50]. Further investigations are necessary to determine what other pathways are involved in the beneficial effects of TSA in VILI-associated pulmonary fibrosis. Additionally, our Akt-deficient mice are Akt heterozygous knockout that induced target mutation in Akt1 gene. The levels of total Akt protein expression were similar between Akt-deficient and wild-type mice because the reduction of Akt1 gene expression was masked by the expression of other isoforms of Akt2 and Akt3 [51–53].

## Conclusions

Our study demonstrated that using the antifibrotic agent HDAC inhibitor TSA can reduce MV-augmented bleomcyin-induced pulmonary fibrosis by reducing alveolar capillary leakage, oxidative stress, MMP-9 and PAI-1, and total collagen through the inhibition of EMT, and achieve pathological, radiological, and functional improvements in our animal model that mimicked the fibroproliferation in ARDS. These beneficial effects are partly mediated through HDAC4-Akt signaling. Our results can be translated into clinical application by using TSA to attenuate early pulmonary fibroproliferation in patients with ARDS requiring MV.

## Supporting information

S1 Table Trichostatin A inhibited MV-augmented bleomycin-induced microvascular leak, lung edema, oxygen radicals, MMP-9 and PAI-1 production. Five days after administering bleomycin, Evans blue dye analysis (A), lung wet-to-dry-weight ratio (B), BAL fluid total protein (C), MDA (D), MMP-9 (E) and PAI-1 (F) secretion in BAL fluid were from the lungs of nonventilated control mice and mice ventilated at a tidal volume of 6 mL/kg (V_T_ 6) or 30 mL/kg (V_T_ 30) for 5 h with room air (n = 5 per group). TSA 2 mg/kg was given intraperitoneally 1 h before mechanical ventilation. Data are presented as mean ± SD.
(XLS)

S2 Table Trichostatin A reduced MV-augmented bleomycin-induced activation of HDAC4. (A) Five days after bleomycin treatment, total HDAC activity was from the lungs of nonventilated control mice and mice ventilated at a tidal volume of 6 mL/kg or 30 mL/kg for 5 h with room air (n = 5 per group). (B and C) Western blots performed using antibodies that recognize HDAC2, HDAC4, and GAPDH expression in lung tissue five days after bleomycin treatment were from nonventilated control mice and mice ventilated at a tidal volume of 6 mL/kg or 30 mL/kg for 5 h with room air (n = 5 per group). (D) Real-time polymerase chain reaction of HDAC4 mRNA and GAPDH mRNA expression in lung tissue after five days of bleomycin treatment were from nonventilated control mice and mice ventilated at a tidal volume of 6 mL/kg or 30 mL/kg for 5 h with room air (n = 5 per group). Arbitrary units were expressed as the ratio of HDAC2 to GAPDH, HDAC4 to GAPDH, and HDAC4 mRNA to GAPDH (n = 5 per group). TSA 2 mg/kg was given intraperitoneally 1 h before mechanical ventilation. Data are presented as mean ± SD.
(XLS)

S3 Table Trichostatin A reduced MV-augmented bleomycin-induced collagen production. Total collagen (A), and hydroxyproline (OH) (B) of lung tissue after five days of bleomycin administration were from nonventilated control mice and mice ventilated at a tidal volume of 6 mL/kg or 30 mL/kg for 5 h with room air (n = 5 per group). TSA 2 mg/kg was given intraperitoneally 1 h before mechanical ventilation. Data are presented as mean ± SD.
(XLS)

S4 Table Trichostatin A suppressed MV-augmented bleomycin-induced collagen gene expression. After five days of bleomycin treatment, real-time polymerase chain reaction of fibronectin (A), MMP-9 (B), and 1a1 (C) mRNA expression were from the lungs of nonventilated control mice and mice ventilated at a tidal volume of 6 mL/kg or 30 mL/kg for 5 h with room air (n = 5 per group). Arbitrary units were expressed as the ratio of fibronectin, MMP-9, and 1a1 mRNA to GAPDH (n = 5 per group). TSA 2 mg/kg was given intraperitoneally 1 h before mechanical ventilation. Data are presented as mean ± SD.
(XLS)

S5 Table Trichostatin A attenuated MV-augmented bleomycin-induced fibrogenic biomarkers. Five days after bleomycin treatment, the fibrotic scoring (A) was quantified as the average number of 10 nonoverlapping fields in Masson’s trichrome staining of paraffin lung sections (n = 5 per group) were from nonventilated control mice and mice ventilated at a tidal volume of 6 mL/kg or 30 mL/kg for 5 h with room air. TSA 2 mg/kg was given intraperitoneally 1 h before mechanical ventilation. Data are presented as mean ± SD.
(XLS)

S6 Table Trichostatin A and Akt heterozygous knockout abrogated MV-augmented bleomycin-induced Akt phosphorylation. (A) Representative micrographs (x400) with phosphorylated Akt staining of paraffin lung sections and quantification 5 days after administering bleomycin were from nonventilated control mice and mice ventilated at a tidal volume of 6 mL/kg or 30 mL/kg for 5 h with room air (n = 5 per group). (B and C) Western blots were performed using an antibody that recognize the phosphorylated Akt expression and antibodies that recognize total Akt, HDAC4, and GAPDH expression in lung tissue five days after bleomycin treatment from nonventilated control mice and mice ventilated at a tidal volume of 6 mL/kg or 30 mL/kg for 5 h with room air (n = 5 per group). Arbitrary units were expressed as the ratio of phospho-Akt to Akt and HDAC4 to GAPDH (n = 5 per group).TSA 2 mg/kg was given intraperitoneally 1 h before mechanical ventilation. Data are presented as mean ± SD.
(XLS)

S7 Table Inhibition of MV-augmented bleomycin-induced lung inflammation and collagen production by Akt heterozygous knockout. Five days after administering bleomycin, Evans blue dye analysis (A), lung wet-to-dry-weight ratio (B), BAL fluid total protein (C), MDA (D), MMP-9 (E) and PAI-1 (F) secretion in BAL fluid, total collagen (G), and hydroxyproline (OH) of lung tissue (H) were from the lungs of nonventilated control mice, and wild type and Akt-deficient mice ventilated at a tidal volume 30 mL/kg for 5 h with room air (n = 5 per group). Data are presented as mean ± SD.
(XLS)

S8 Table Reduction of MV-augmented bleomycin-induced collagen gene expression and epithelial-mesenchymal transition in Akt-deficient mice. After five days of bleomycin treatment, real-time polymerase chain reaction of fibronectin (A), MMP-9 (B), and 1a1 (C) mRNA expression were from the lungs of nonventilated control mice, and wild-type and Akt-deficient mice ventilated at a tidal volume of 30 mL/kg for 5 h with room air (n = 5 per group). Arbitrary units were expressed as the ratio of fibronectin, MMP-9, and 1a1 mRNA to GAPDH (n = 5 per group). The fibrotic scoring (D) was quantified as the average number of 10 nonoverlapping fields in Masson’s trichrome staining of paraffin lung sections (n = 5 per group) five days after administering bleomycin were from nonventilated control mice, and wild type and Akt-deficient mice ventilated at a tidal volume of 30 mL/kg for 5 h with room air. Data are presented as mean ± SD.
(XLS)

## Abbreviations

Akt: serine/threonine kinase/protein kinase B
ALI: acute lung injury
ARDS: acute respiratory distress syndrome
BAL: bronchoalveolar lavage
ECM: extracellular matrix
EBD: Evans blue dye
EMT: epithelial-mesenchymal transition
HDAC: histone deacetylases
IPF: idiopathic pulmonary fibrosis
MAPK: mitogen-activated protein kinase
μCT: micro-computer tomography
MMP-9: matrix metalloproteinase-9
MV: mechanical ventilation
PAI-1: plasminogen activator inhibitor-1
PCR: polymerase chain reaction
α-SMA: α-smooth muscle actin
TEM: transmission electron microscopy
TGF-β1: transforming growth factor-β1
TSA: trichostatin A
VILI: ventilator-induced lung injury
V_T_: tidal volume
